# Supplementary material for: Hypothermic Oxygenated Machine Perfusion (HOPE) Prior to Liver Transplantation Mitigates Post-Reperfusion Syndrome and Perioperative Electrolyte Shifts
Source: J Clin Med. 2022 Dec 12;11(24):7381. doi: 10.3390/jcm11247381 (PMC9786550; doi:10.3390/jcm11247381)
Supplement: Supplementary file 1 [file jcm-11-07381-s001.zip › jcm-2035149-supplementary.pdf]

Supplementary Materials

Table S1: Description of liver transplant recipients' comorbidities defined as relevant for further statistical evaluation.

Relevant recipient comorbidities at date of liver transplantation	
**Cardiovascular**	
	Arterial Hypertension
	Arterial Fibrillation
	Coronary Artery Disease
	Cardiomyopathy
	Cerebral Vascular Disease
	Arrhythmias
	Heart Valve Disease
**Respiratory**	
	Asthma bronchiale
	Nicotine Abuse ≥ 25py
**Renal**	
	Chronic Renal Insufficiency (KDIGO CKD stages ≥ 3)
**Endocrine / Metabolic**	
	Diabetes mellitus
	Obesity (BMI > 30)
	Cachexia (BMI < 17)
	Adrenal Insufficiency
	Hypothyreoidism
	Grave's Disease
	Dyslipidemia
**Gastrointestinal**	
	Infectious Bowel Disease
	Chronic Pancreatitis
**Neurological**	
	Epilepsy
	Multiple Sclerosis
	Polyneuropathy
**Hematopoetic**	
	Bone Marrow Deficiency
	Hereditary Coagulopathy
**Other**	
	Rheumatism
	Sarcoidosis
	IVDU

BMI: Body Mass Index; CKD: Chronic Kidney Disease; IVDU: intravenous drug use; KDIGO: "Kidney; Disease: Improving Global Outcomes"; py: pack years.

Table S2: Description of liver transplant recipients' transplant indications, previously defined as "Other".

Indications for transplant defined as "Other" at date of liver transplantation	All, N (%)	Liver Graft Preservation Techniques		p-Value (HOPE vs. SCS)
		-SCS- **N (%)**	***-HOPE-*** **N (%)**	
**Other**				0.99
Sarcoidosis	1 (1.0%)	0 (0%)	1 (2.0%)	
ITBL	5 (5.0%)	3 (6.0%)	2 (4.0%)	
Chronic Graft Failure	1 (1.0%)	0 (0%)	1 (2.0%)	
Recurrent Cholangiosepsis	2 (2.0%)	0 (0%)	2 (4.0%)	
α-1-antitrypsin Deficiency	1 (1.0%)	0 (0%)	1 (2.0%)	
Acute Graft Failure	1 (1.0%)	0 (0%)	1 (2.0%)	
Post-Kasai Cirrhosis	2 (2.0%)	1 (2.0%)	1 (2.0%)	
Porphyria	1 (1.0%)	0 (0%)	1 (2.0%)	
SSC	1 (1.0%)	0 (0%)	1 (2.0%)	
ADPKD	1 (1.0%)	1 (2.0%)	0 (0%)	
IPBN	1 (1.0%)	1 (2.0%)	0 (0%)	
Wilson's Disease	1 (1.0%)	1 (2.0%)	0 (0%)	
Budd-Chiari Syndrome	1 (1.0%)	1 (2.0%)	0 (0%)	
PNF	2 (2.0%)	2 (4.0%)	0 (0%)	
**Total**	21 (21.0%)	10 (10.0%)	11 (11.0%)	

ADPKD: autosomal dominant polycystic kidney disease; HOPE: Hypothermic Organ Perfusion; IPBN: Intraductal Papillary Neoplasm of the Bile Duct; ITBL: Ischemic Type Biliary Lesion; PNF: Primary Non-Function; SCS: Static Cold Storage; SSC: Secondary Sclerosing Cholangitis.
